# Patient-Specific Variability in Interleukin-6 and Myeloperoxidase Responses in Osteoarthritis: Insights from Synthetic Data and Clustering Analysis

**DOI:** 10.3390/jpm15010017

**Published:** 2025-01-04

**Authors:** Laura Jane Coleman, John L. Byrne, Stuart Edwards, Rosemary O’Hara

**Affiliations:** 1 Department of Applied Science, South East Technological University, R93 V960 Carlow, Ireland; john.byrne@setu.ie (J.L.B.); rosemary.ohara@setu.ie (R.O.); 2 UPMC Aut Even Hospital, R95 D370 Kilkenny, Ireland; orthojoints.auteven@umpc.ie

**Keywords:** osteoarthritis (OA), fibroblast-like synoviocytes (FLS), lipopolysaccharide (LPS), interleukin-6 (IL-6), myeloperoxidase (MPO), hierarchical clustering (HC), synthetic data

## Abstract

**Objectives:** This study investigated the inflammatory responses of fibroblast-like synoviocytes (FLS) isolated from osteoarthritis (OA) patients, stimulated with lipopolysaccharide (LPS) and interleukin-6 (IL-6). Both experimental and synthetic data were utilised to investigate the variability in IL-6 and myeloperoxidase (MPO) production and its implications for OA pathogenesis. **Methods:** Synovial biopsies were obtained from OA patients undergoing joint replacement surgery. FLS were isolated, cultured, and stimulated with varying concentrations of LPS and IL-6. The production of IL-6 and MPO was measured using enzyme-linked immunosorbent assays (ELISA). Synthetic data generation techniques expanded the dataset to support comprehensive statistical analyses. **Results:** The patterns of inflammatory responses revealed distinct patient subgroups, highlighting individual variability. The integration of synthetic data with experimental observations validated their reliability and demonstrated dose-dependent differences in IL-6 and MPO production across patients. **Conclusions:** The results highlighted the importance of patient-specific factors in OA inflammation and demonstrated the utility of combining experimental and synthetic data to model individual variability. The results support the development of personalised treatment strategies in OA. Future research should include larger patient datasets and an exploration of molecular mechanisms underlying these responses.

## 1. Introduction

Osteoarthritis (OA) is a debilitating joint disease characterised by articular cartilage degradation and inflammation, leading to structural changes within the joints. Fibroblast-like synoviocytes (FLS) are essential in understanding the complexities of OA [1], as they are active contributors to disease progression [2,3,4]. FLS are located within the synovial membrane and help maintain joint function. However, in OA, these cells unleash matrix-degrading enzymes and pro-inflammatory mediators, resulting in joint destruction [2].

Numerous studies have explored FLS in OA and rheumatoid arthritis (RA) to better understand their roles in these inflammatory joint diseases. A study conducted in 2019 isolated FLS from the synovial tissues of twenty-two patients, evenly divided between OA and RA. Both groups exhibited consistent fibroblast morphology and similar profiles of cell surface antigens. The study evaluated the cellular response to the pro-inflammatory cytokine tumour necrosis factor-alpha (TNF-α) and the anti-inflammatory drug methotrexate (MTX), highlighting FLS as potential therapeutic targets in OA and RA [5].

Notably, a study by José Alcaraz et al. explored the inflammatory responses of FLS to various stimuli, highlighting the significance of these cells in disease progression [3]. Similarly, research focused on the molecular mechanisms underlying FLS activity in OA, providing critical insights into potential therapeutic targets [4]. Another study examined the impact of cytokine environments on FLS behaviour, offering valuable data that align with the current research aims. These studies underscore the relevance and necessity of further investigations into FLS, reinforcing the importance of this research [1].

Culturing primary cells like FLS is demanding due to their finite lifespan in vitro, which poses a significant limitation [6]. Cell culturing is used to study cell physiology and biochemistry, drug effects, mutagenesis, carcinogenesis, and more. Culture conditions, which are cell type-dependent, typically require a medium with essential nutrients, growth factors, hormones, and gases and a physiochemically regulated environment. Recognising cell growth phases—lag, log, stationary, and death—is crucial for assessing changes due to growth or contamination [7,8,9]. In OA, variability in inflammatory responses arises from patient-specific factors such as genetics, comorbidities, and medical history. These factors complicate the development of universal treatment protocols and highlight the need for personalised therapeutic approaches [10,11]. The modelling approach addresses this need by enabling individualised treatment strategies that are tailored to patient-specific profiles. The generation of synthetic data has been increasingly employed in biomedical research to expand datasets, address constraints in primary cell cultures, and improve statistical power, especially when dealing with small sample sizes [12,13,14].

Integrating synthetic data to complement experimental approaches, this study aimed to provide a reliable investigation of FLS responses to inflammatory stimuli, specifically lipopolysaccharide (LPS) and interleukin-6 (IL-6) [12,15]. The use of synthetic data in combination with experimental observations ensures a comprehensive analysis, addressing inter-patient variability and providing a robust foundation for advancing personalised therapeutic strategies.

Synthetic data generated through generative adversarial networks (GANs), foundational AI-driven methods, enhance the robustness of small datasets by expanding sample sizes and improving statistical power. However, careful validation is necessary to avoid biases [13,16]. AI methodologies, including clustering techniques, such as hierarchical clustering and t-distributed stochastic neighbour embedding (t-SNE), are effective in identifying subgroups within heterogenous datasets [15]. Validation methods such as k-nearest neighbour (kNN) classification and ROC analysis, both standard tools in machine learning, are essential for confirming the biological relevance of synthetic data and their alignment with experimental observations. These tools collectively ensure meaningful insights into OA pathogenesis while leveraging the strengths of modern AI and machine learning techniques [17,18].

FLS produce various pro-inflammatory substances, including inflammatory cytokines, nitric oxide (NO), and prostaglandin E_2_ (PGE_2_), which are closely linked to OA symptoms such as joint pain, swelling, and disease progression [2]. Analysing cytokine stimulation effects on FLS behaviour in patients with hip and knee joint conditions aimed to uncover patterns and trends that contribute to the understanding of the disease process.

In 2022, a study examined the role of hyaluronic acid (HA) in joint inflammation, focusing on FLS within the context of OA. The study revealed that co-cultures of RA FLS with activated CD4 T cells generated an HA-enriched extracellular matrix (ECM), enhancing monocyte adhesion, which is a crucial step in inflammation [1]. Both OA and RA FLS co-cultures with activated CD4 T cells exhibited an increased expression of inflammatory cytokines, highlighting the significance of an HA-enriched ECM in creating a pathogenic microenvironment. These findings provided a basis for exploring how FLS-HA interactions contributed to OA pathogenesis and aligned with the objectives of this research.

This study aimed to isolate FLS from tissue biopsies to gain insights into their behaviour in the context of OA. Specifically, it addressed patient-specific inflammatory responses to advance therapeutic approaches. Despite ongoing research, traditional experimental studies often contend with limitations imposed by small sample sizes and inter-patient variability, which can obscure significant biological insights. Machine learning techniques, such as clustering algorithms, provide a promising solution by uncovering patterns in complex datasets. However, their effectiveness is often constrained by small dataset sizes, which can be mitigated through the generation of validated synthetic data [19].

Integrating synthetic data with experimental observations provided a novel approach for investigating the responses of fibroblast-like synoviocytes (FLS) to inflammatory stimuli in OA [15]. This study provided three significant contributions. First, it identified distinct patient subgroups using advanced clustering techniques, which underscored the heterogeneity in inflammatory responses. Second, it validated the biological relevance of synthetic data through machine learning tools such as kNN classification and ROC analysis, ensuring alignment with experimental findings. Finally, it established a robust framework for exploring dose–response variability and its implications for advancing personalised medicine strategies in OA treatment [20,21]. Together, these contributions aimed to address challenges in small datasets and patient-specific variability, paving the way for novel therapeutic approaches.

## 2. Methods

### 2.1. Patient Recruitment and Sample Preparation

#### 2.1.1. Ethical Considerations

Ethical approval for this project was obtained from the South East Technological University (SETU) Ethics committee and Aut Even Hospital Kilkenny (protocol code 160 and date of approval 8 December 2016). Informed consent and health screening questionnaire responses were obtained from patients prior to participation in this study. 

#### 2.1.2. Patients

The study demographic included patients with knee osteoarthritis (KOA) and hip osteoarthritis (HOA) undergoing total knee replacement (TKR) and total hip replacement (THR) surgeries. The mean age ± SD of the patient group (*n* = 7) was 73.14 ± 9.08 years. Each participant in this study was randomly assigned a unique number for identification purposes. Synovial membrane biopsies were obtained with informed consent from patients with OA (*n* = 7) during knee (*n* = 5) and hip (*n* = 2) replacement surgeries.

Patients with severe end-stage OA referred to those who required joint replacement surgery. This classification was based on diagnosis by an orthopaedic surgeon using radiographic features and scales such as the Kellgren–Lawrence grading system, specifically grades three and four, which are characterised by significant joint space narrowing, large osteophytes, and significant bone deformity [22,23]. Patients with end-stage OA were defined as those having exhausted all other treatments with no relief, making joint replacement surgery the last option.

#### 2.1.3. Synovial Biopsy Processing

Synovial membrane biopsies were obtained from patients undergoing knee or hip replacement surgery. These biopsies were selected based on visual inspection by the orthopaedic surgeon at the time of joint replacement. If synovial hypertrophy was present, the synovium was sampled, or in the absence of hypertrophy, the biopsies were randomly chosen [24]. Tissue samples were collected and preserved in RPMI media supplemented with 10% heat-inactivated Foetal Calf Serum (FCS), HEPES, penicillin–streptomycin, and amphotericin. This was transported at −20 °C.

### 2.2. Cell Isolation, Culturing, and Stimulation

#### 2.2.1. Isolation and Culturing of Fibroblast-like Synoviocytes

Under aseptic conditions, primary tissues were enzymatically digested with 1 mg/mL collagenase type 1 (GIBCO, Thermo Fisher Scientific, Paisley, UK) in RPMI 1640 containing L-glutamine (GIBCO, Thermo Fisher Scientific, Paisley, UK) to obtain FLS. The biopsies were incubated for 4 h at 37 °C in a 5% CO_2_ atmosphere in a Memmert CO_2_ incubator (Lennox, Dublin, Ireland). After centrifugation at 2500× *g* for 5 min in a Hettich Rotanta 460RF centrifuge, the supernatants were removed before adding 2 mL of pre-warmed supplemented RPMI-1640. The pellet was vortexed (Vortex-Mixer VM-10) and centrifuged at 2500× *g* for 5 min. The supernatant was discarded, and another 2 mL of media was added to the pellet and vortexed. The dissociated cells were then placed in 5 mL supplemented RPMI in 25 cm^3^ tissue culture flasks. The synovial fibroblast-like synoviocytes were allowed to reach 95% confluency over 10–15 days at 37 °C in a 5% CO_2_ atmosphere.

#### 2.2.2. Trypsinisation and Subculturing of Cultured Adherent Cells

FLS were harvested with 0.25% trypsin and used between the third and sixth passages, as they were found to be a morphologically homogenous fibroblast-like synoviocyte population under a microscope (BMS Inverted biological microscope 74575) [21]. FLSs were subcultured in 25 cm^3^ tissue culture flasks (T25) by washing them with 5 mL of sterile phosphate-buffered saline (1X PBS) to remove any traces of foetal bovine serum (FBS), as it is a trypsin inhibitor. A total of 3 mL of 1X trypsin was added and incubated for 15 min at 37 °C. Cells were only exposed to trypsin for a short time, due to extended exposure damaging the cell’s surface receptors [25]. Adherent cells required a longer exposure time to trypsin than semi-adherent cells [26]. Detached cells were resuspended in supplemented RPMI, and cell viability was determined using Trypan Blue [27].

#### 2.2.3. Stimulation of Fibroblast-like Synoviocytes

The FLS were grown to confluency, and before stimulation, primary cultures were incubated overnight in serum-free media [21]. FLS were then treated with various concentrations of LPS (1000 pg/mL, 10,000 pg/mL, and 100,000 pg/mL) (Merck, Dublin, Ireland) or IL-6 (1000 pg/mL, 10,000 pg/mL, and 100,000 pg/mL) (Peprotech, London, UK) in serum-free media for 3, 6, 9, and 24 h [28]. After incubation, the FLS supernatants were harvested to analyse myeloperoxidase (MPO) and IL-6 production measured by ELISA [29].

#### 2.2.4. Cryopreservation of Adherent Cells

Fibroblast-like synoviocytes (FLS) were cryopreserved during the logarithmic growth phase to ensure cell viability. Pre-confluent cultures were prepared by refreshing the media 24 h before freezing. Cell culture media were removed, and cells were washed with 5 mL of serum-free phosphate-buffered saline (PBS). FLS were trypsinised to mitigate stress factors associated with cryopreservation, such as protease activation leading to molecular-based apoptotic cell death; trypsin was inactivated by incubation at 70 °C for 15 min [30]. The FLS suspension was subsequently transferred to a tissue culture tube containing fetal bovine serum (FBS) medium and centrifuged for 5 min at 300× *g*.

Cells were cryopreserved using either Biofreeze, a broad-spectrum cryoprotectant, or 90% RPMI and 10% dimethyl sulphoxide (DMSO), ensuring the maximum FLS recovery post-thaw [31]. The cryopreservation process was conducted gradually. FLS were frozen at −80 °C overnight and then transferred to the vapour phase of the liquid nitrogen vessel for long-term storage [32].

#### 2.2.5. Enzyme-Linked Immunosorbent Assay

IL-6 and MPO levels in the supernatants were measured using commercially available ELISA kits (Biolegend, London, UK), according to the manufacturer’s instructions. Then, 96-well plates were coated with capture antibodies specific to the target proteins. The samples were added to the wells and incubated for a specified time period. After washing, detection antibodies and substrates were added. Absorbance was measured at 450 nm using a microplate reader. All assays were performed in duplicate, and the mean values were used for analysis.

### 2.3. Synthetic Data Generation and Validation

#### 2.3.1. Synthetic Data Utilisation

Synthetic data were generated using EasyNN-plus (Neural Planner Software Ltd., version 7.0e, Cheadle, UK) to address the limitations of small datasets, employing controlled variance introduction to stimulate realistic patient-specific inflammatory responses. Synthetic data generation has been increasingly utilised in medical research to improve the robustness of datasets, particularly when dealing with limited or incomplete data [13,16]. Recognising the potential risks associated with poorly generated synthetic data, a systematic approach was adopted to ensure alignment with the underlying distributions of real patient data.

Validation techniques, including hierarchical clustering, t-distributed stochastic neighbour embedding (t-SNE), k-nearest neighbour (kNN), and receiver operating characteristic (ROC) curve analysis, ensured the synthetic data accurately represented patient-specific variability [21,33]. These methods mitigated the risk of misleading statistical conclusions and ensured the synthetic data were suitable for robust analyses of dose–response patterns and inter-patient variability [34,35]. By expanding the dataset, synthetic data facilitated a deeper understanding of inflammatory responses, overcoming the limitations imposed by the small real dataset.

#### 2.3.2. k-Nearest Neighbour Analysis

k-nearest neighbour (kNN) analysis, a non-parametric classification regression tool, was used to classify data points based on similarity to a known dataset. Data were first organised into clusters via hierarchical clustering, and each new point was classified by comparing each unknown to its “k”-nearest neighbour. The optimal “k” value maximises the gap, indicating a clear clustering structure. For each new point, the majority class among the nearest neighbour determines its classification, ensuring robust model performance. This approach validated the alignment of synthetic data with real patient responses. The k-NN method is widely applied in biomedical studies to enhance diagnostic accuracy [17].

#### 2.3.3. Receiver Operating Characteristic Curves

Receiver operating characteristic (ROC) curves evaluated classification model performance by plotting true positive rates against false positive rates at various threshold values. The area under the curve (AUC) quantified accuracy, with higher AUC values indicating better model performance [18]. ROC analysis validated the reliability of k-NN classifications in distinguishing between inflammatory response categories.

### 2.4. Statistical Analysis and Clustering Analysis

Statistical analysis was performed using Jamovi Version 2.3.28 [Copyright 2021]. These tools provided insights into data patterns, enhancing the interpretations of the analyses [36,37].

#### 2.4.1. Normality and Group Comparisons

The normality of data was assessed using the Shapiro–Wilk test. This test is commonly used for validating the assumption of normality in datasets and has been detailed in recent statistical research [38]. Non-parametric methods, including the Kruskal–Wallis test, were used to analyse differences across groups. This method has been recently applied in COVID-19 data analysis [39].

Post hoc pairwise comparisons were performed using the Dwass–Steel–Critchlow–Fligner (DSCF) pairwise comparison method to identify significant differences between specific groups while controlling for the type I error rate, making it suitable for biomarker data with non-normal distributions. The application of the DSCF test provided detailed insights into pairwise differences in IL-6 and MPO production across conditions. This method has been effectively utilised in recent biomedical research to compare groups in non-parametric datasets [40].

#### 2.4.2. Hierarchical Clustering

Hierarchical clustering was used to categorise data points into clusters based on similarity, helping to identify potential disease subtypes. Hierarchical clustering generates a dendrogram, a tree-like structure where data points are grouped based on calculated distances, forming clusters that represent similar characteristics. This technique has been instrumental in multi-omics research, where clustering across biological layers enhances the understanding of disease progression and patient-specific treatment needs [34].

Ward.D2 was used for constructing dendrograms, minimising within-cluster variance by squaring dissimilarities for optimal clustering. Clustering accuracy was assessed using the cophenetic correlation coefficient, ensuring the dendrogram preserved the original pairwise distances. These methods have been shown to enhance classification power and improve disease predictions, particularly in complex datasets [41]. The optimal number of clusters was identified using the gap statistic, which compares the total intra-cluster variation across different values of k. The clustering structure was assessed to ensure that the selected k deviated from a uniform distribution, with Gap (k) values indicating optimal clustering points.

#### 2.4.3. t-Distributed Stochastic Neighbour Embedding

t-distributed stochastic neighbour embedding (t-SNE) is a non-linear dimensionality reduction technique used to visualise complex, high-dimensional data. Unlike Principal Component Analysis (PCA), which uses linear transformation, t-SNE captures both local and global data structures by mapping data points into a lower-dimensional space while retaining cluster relationships [42]. t-SNE employs Kullback–Leibler (KL) divergence to optimise data positioning in low-dimensional space, making it suitable for complex, multidimensional datasets [20]. For this study, t-SNE enhanced the visualisation of data clusters, supporting a clear understanding of patient-specific inflammatory responses.

## 3. Results

### 3.1. Variability in Inflammatory Responses

This study evaluated the variability in IL-6 and MPO production by FLS from OA patients in response to LPS and IL-6 stimulation. By integrating experimental and synthetic data, the analysis provided a comprehensive evaluation of the dose–response relationships and patient-specific differences in inflammatory responses. These findings highlighted the heterogeneity among patients, with variability influenced by factors such as immune thresholds, medication use, and underlying health conditions.

Initial normality and group comparisons using the Shapiro–Wilk and Kruskal–Wallis tests assessed the distributions and identified significant differences between groups. Post hoc analyses further described group-specific trends in IL-6 and MPO production. Hierarchical clustering and t-SNE dimensionality reduction identified distinct patient subtypes, uncovering patterns of inflammatory responses that extended beyond individual-level observations. These analyses revealed three primary subgroups characterised by varying sensitivities to LPS and IL-6 stimuli, underscoring the heterogeneity of OA-associated inflammatory responses.

The synthetic data, validated by k-nearest neighbour (kNN) classification and ROC curve analysis, closely aligned with real patient responses, supporting model reliability. This validation strengthened this study’s findings, providing a robust framework for investigating patient-specific treatment implications and supporting the potential for personalised therapeutic strategies.

### 3.2. Dose–Response Patterns in IL-6 and MPO Production

Variability in IL-6 production was evident across patients. Patient N2 required a higher concentration (100,000 pg/mL) to elicit a marked response, whereas patients N6, N9, and N10 exhibited elevated responses even at 1000 pg/mL. In contrast, patients N4, N5, and N8 exhibited gradual, gradient increases in IL-6 levels across LPS concentrations, consistent with a more linear response profile.

For MPO production, similar inter-patient variability was observed. Patient N2 exhibited a peak response at high LPS concentrations (100,000 pg/mL), whereas other patients such as N6, N8, and N10 responded at lower concentrations (1000–10,000 pg/mL). Patients with comorbidities, such as high blood pressure or diabetes, showed minimal MPO responses, potentially due to the influence of these conditions on inflammatory thresholds.

Synthetic data expansions using data jitter validated these trends by replicating individual and cohort responses, reinforcing the robustness of the observed patterns. These findings emphasised the importance of patient-specific factors in shaping inflammatory responses.

#### 3.2.1. Normality and Group Comparisons

Shapiro–Wilk tests confirmed that the data were non-parametric (W = 0.664–0.935, *p* (*p*-value) < 0.001). Despite the relatively high W values, which suggest a closer fit to a normal distribution, the statistically significant *p*-values indicated substantial deviations from normality [43]. This finding validated the use of non-parametric statistical methods, such as the Kruskal–Wallis test, for subsequent analyses.

The Kruskal–Wallis test, using stimulant concentrations as the grouping variable, revealed significant differences in IL-6 production across varying LPS concentrations (χ^2^ = 3049, df = 3, *p* < 0.001, ε^2^ = 0.268), with approximately 26.8% of the variance attributed to LPS dose differences (Table 1). Similarly, MPO production showed significant variability in response to both LPS (χ^2^ = 435, df = 5, *p* < 0.001, ε^2^ = 0.0397) and IL-6 concentrations (χ^2^ = 656, df = 4, *p* < 0.001, ε^2^ = 0.0577). These results indicated that both stimulants significantly influenced MPO production, though with smaller effect sizes compared to IL-6 production in response to LPS.

To gain further insights into individual patient responses, additional Kruskal–Wallis tests were conducted with patients as the grouping variable (Table 2), allowing for the exploration of individual variability in biomarker levels. IL-6 production in response to LPS varied significantly among patients (χ^2^ = 6332, df = 6, *p* < 0.001, ε^2^ = 0.557), as did MPO production in response to both LPS (χ^2^ = 9756, df = 7, *p* < 0.001, ε^2^ = 0.890) and IL-6 (χ^2^ = 10,149, df = 6, *p* < 0.001, ε^2^ = 0.893) (Table 2). These high effect sizes indicated that a significant proportion of MPO variability could be attributed to individual patient factors, highlighting the impact of patient-specific characteristics.

#### 3.2.2. Post Hoc Analysis for Group Differences

To further investigate the differences highlighted by the Kruskal–Wallis tests, pairwise comparisons were conducted using the Dwass–Steel–Critchlow–Fligner (DSCF) test. This analysis confirmed the statistical significance of these differences. For IL-6 production, significant differences were observed between 10,000 pg/mL and the control (W = −58.1, *p* < 0.001), as well as between 100,000 pg/mL and the control (W = −650, *p* < 0.001), confirming that IL-6 production increases significantly with higher LPS concentrations.

For MPO production in response to LPS, the DSCF test identified significant differences between 10,000 pg/mL and 100,000 pg/mL (W = 27.2, *p* < 0.001) and between 100,000 pg/mL and the control (W = −33.8, *p* < 0.001). This further supported the trend of increased MPO production with higher LPS concentrations.

Similarly, MPO response to IL-6 revealed significant pairwise differences, including those between 10,000 pg/mL and 100,000 pg/mL (W = 10.5, *p* < 0.001) and between 100,000 pg/mL and the control (W = −6.2, *p* < 0.001). However, comparisons, such as that between 100,000 pg/mL and 1000 pg/mL (W = −3.7, *p* = 0.070) and that between 1000 pg/mL and the control (W = −2.9, *p* = 0.240), were not statistically significant, indicating that MPO response thresholds vary across different concentrations.

When grouped by patients, the DSCF test demonstrated substantial individual variability, with highly significant W values (all *p* < 0.001) across comparisons. These results underscored the substantial impact of patient-specific factors on MPO production, particularly in response to IL-6, highlighting considerable heterogeneity among individual patients.

### 3.3. Identification of Patient Subgroups Through Clustering

**Figure 1:**
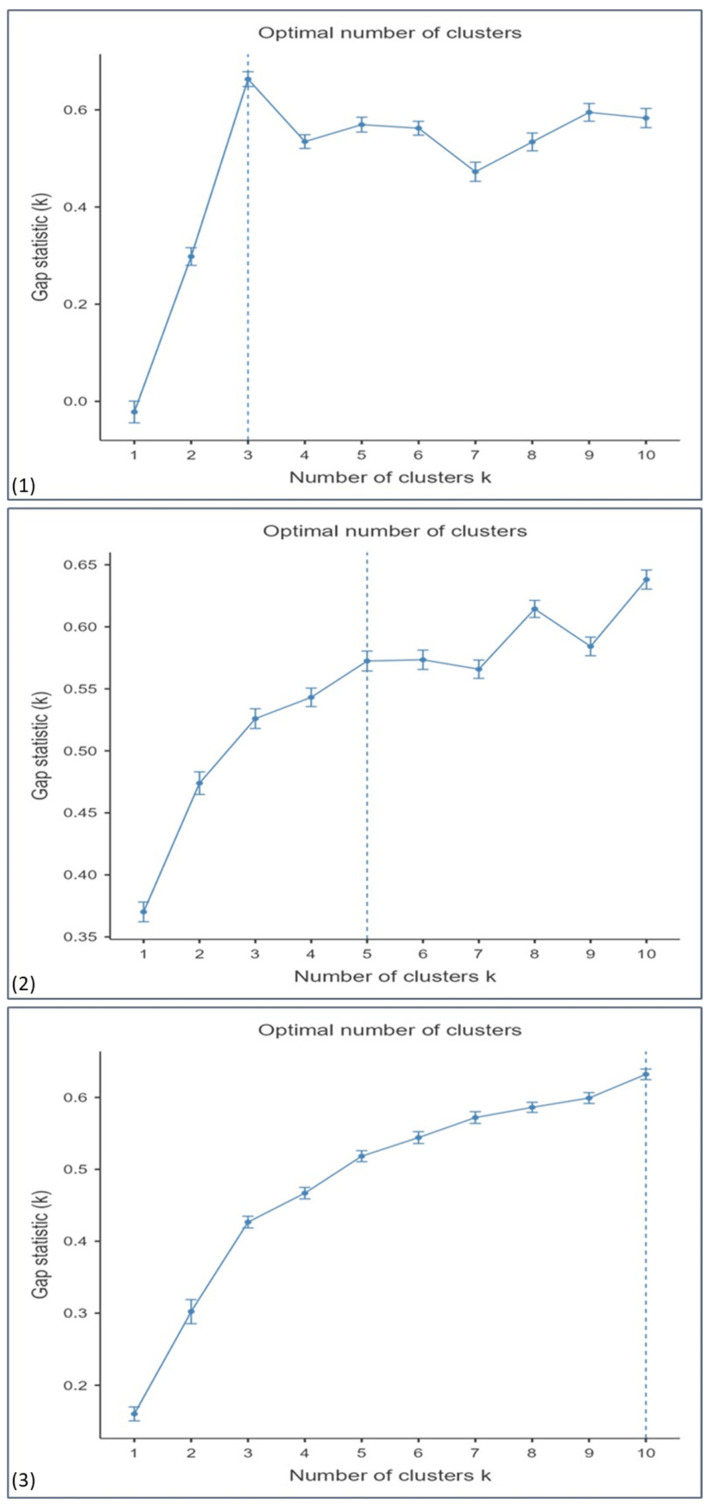
Optimal number of clusters determined for varying stimulant concentrations in different analyses. (**1**) LPS/IL-6 analysis, (**2**) LPS/MPO analysis, and (**3**) IL-6/MPO analysis, displaying optimal cluster number for different stimulant conditions.

**Figure 2:**
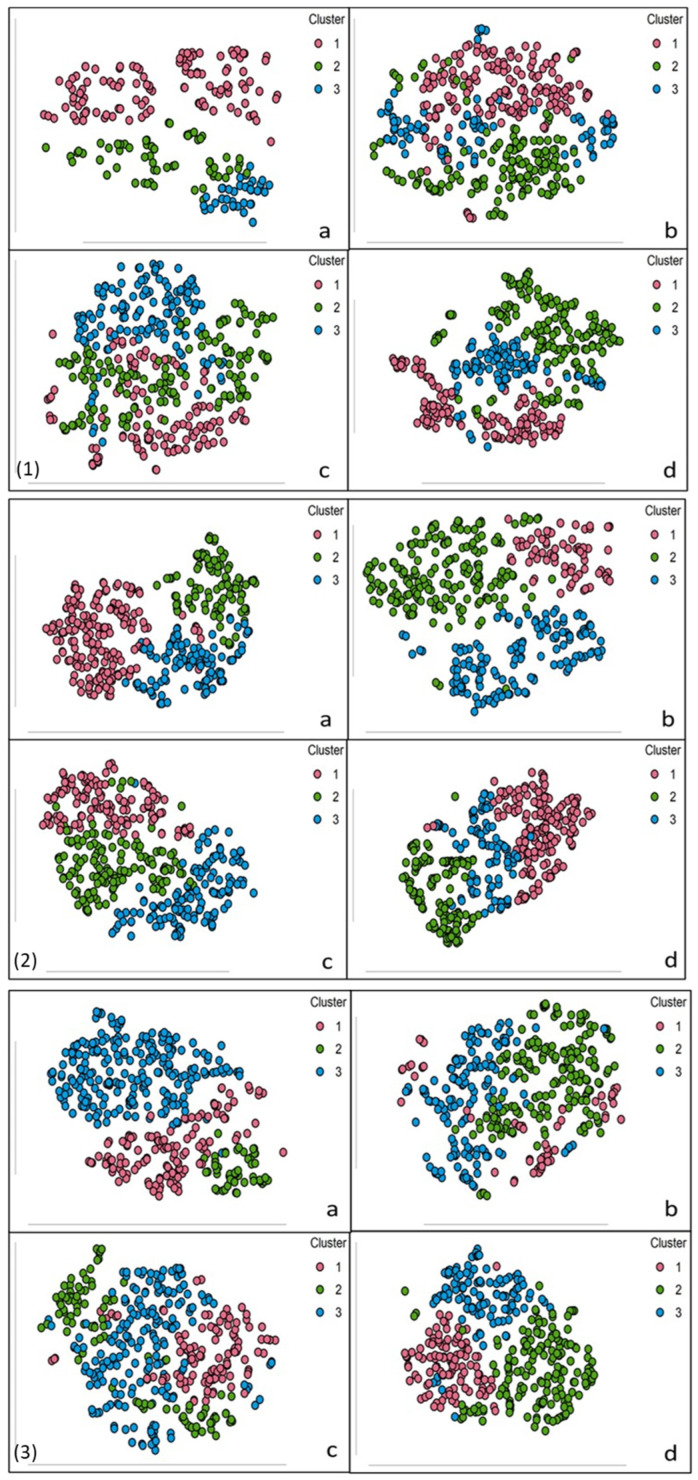
t-SNE cluster plots showing three distinct clusters at varying stimulant concentrations for different analyses. (**1**) LPS/IL-6 analysis, (**2**) LPS/MPO analysis, and (**3**) IL-6/MPO analysis with stimulant concentrations of (**a**) control, (**b**) 1000 pg/mL, (**c**) 10,000 pg/mL, and (**d**) 100,000 pg/mL.

**Figure 3:**
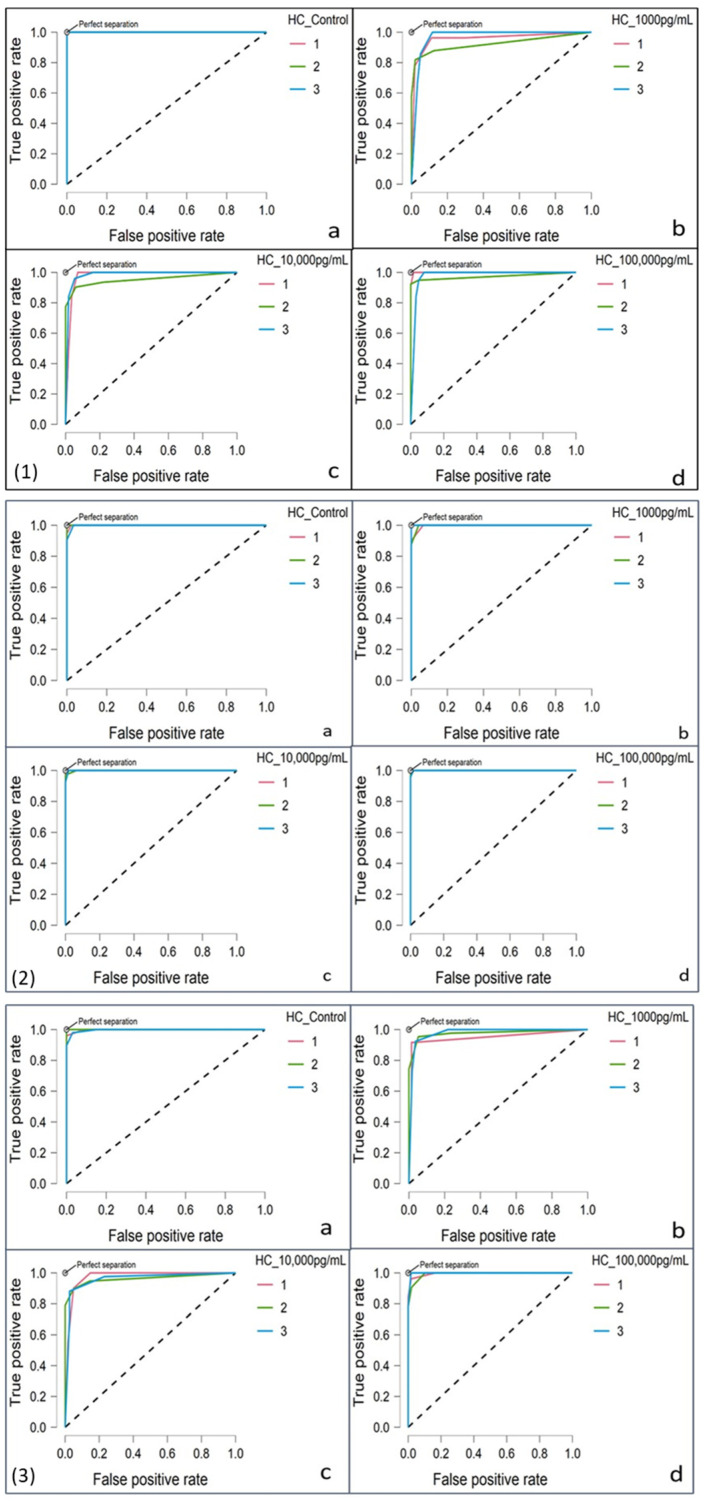
Receiver operating characteristic (ROC) curves for different analyses at varying stimulant concentrations, with clusters 1, 2, and 3 representing groups identified through confusion matrix analysis. (**1**) LPS/IL-6 analysis, (**2**) LPS/MPO analysis, and (**3**) IL-6/MPO analysis with stimulant concentrations of (**a**) control, (**b**) 1000 pg/mL, (**c**) 10,000 pg/mL, and (**d**) 100,000 pg/mL. The dotted diagonal line represents the line of no discrimination (AUC = 0.5), serving as a baseline for random classification performance.

Hierarchical clustering identified optimal cluster numbers for each analysis with three clusters for LPS/IL-6 analysis, five for LPS/MPO, and ten for IL-6/MPO (Figure 1). However, high-order cluster effects meant higher complexity. Therefore, three clusters were fixed to improve visual clarity and biological relevance.

t-SNE plots (Figure 2) confirmed three distinct clusters under varying stimulant concentrations: (a) control, (b) 1000 pg/mL, (c) 10,000 pg/mL, and (d) 100,000 pg/mL. Clusters were most distinct at 100,000 pg/mL, highlighting variability in inflammatory thresholds. The gap statistic (>0.4) validated the three-cluster model, ensuring biological relevance while avoiding unnecessary complexity (Figure 3).

### 3.4. Validation of Synthetic Data

The synthetic data, validated using k-nearest neighbour (kNN) classification and ROC curve analysis, closely aligned with real patient responses.

#### 3.4.1. kNN Classification

Synthetic data were generated for each of the LPS/IL-6, LPS/MPO, and IL-6/MPO datasets, producing 400 data points per variable and a total of 3200 data points for each dataset. kNN classification assessed dose–response patterns, identifying whether they were uni-modal or multi-modal, aiming to identify patient-specific response profiles to LPS and IL-6 stimulation.

Across all datasets, kNN classification consistently revealed multi-modal response patterns, suggesting variability in patient responses rather than uniform trends. Classification accuracies were robust, with values ranging from 0.877 to 0.988, with controls showing minor variations across datasets but consistently high accuracy. At lower concentrations (1000 pg/mL), accuracy was 0.877, highlighting variability in early responses. Accuracy improved at intermediate concentrations (10,000 pg/mL, 0.901) and reached its peak at 100,000 pg/mL (0.951), demonstrating the model’s ability to distinguish dose–response effects at the highest stimulant concentration.

Decision Boundary Matrix (DBM) analyses were used to visualise the response modes from kNN classification. Differences in sensitivity to inflammatory stimuli were observed across samples, with initial peaks representing baseline inflammatory responses and subsequent peaks reflecting multi-modal patterns at higher stimulant concentrations.

For example, patient N2, with no reported medication, exhibited consistent high-threshold responses, with distinct peaks at the highest LPS concentration (100,000 pg/mL). Conversely, patient N10, with comorbidities such as hypertension and diabetes, displayed lower thresholds and multi-modal peaks across all concentrations, suggesting potential susceptibility to prolonged inflammatory states. These observations underscored the role of patient-specific factors in shaping inflammatory responses, validating kNN findings and the robustness of the classification model in capturing heterogeneity.

#### 3.4.2. ROC Curve Analysis

ROC curve analysis further validated the clustering model’s efficiency and reliability of cluster arrangements, with area under the curve (AUC) values exceeding >0.7 across all datasets, demonstrating strong model performance, showing clear separation from the baseline (Figure 3).

Confusion matrix analyses revealed distinct patterns in cluster dominance across varying stimulant concentrations. For controls, Cluster 1 was the most prevalent in LPS/IL-6 and LPS/MPO analyses (0.58 and 0.43, respectively), while Cluster 3 dominated in IL-6/MPO analysis (0.59). At 1000 pg/mL, Cluster 2 was the most dominant across all datasets (0.33 and 0.4 in LPS/IL-6 and LPS/MPO, respectively), maintaining its prominence at 10,000 pg/mL (0.35). At 100,000 pg/mL, Cluster 2 remained prominent, with varying contributions from Clusters 1 and 3 depending on the analysis.

The consistency of clustering across the stimulant concentrations reinforced the model’s reliability in capturing patient-specific variability in inflammatory responses. These finding provided critical insights into inflammatory dynamics and highlighted the potential for data-driven stratification in personalised medicine.

## 4. Discussion

This study underscored the heterogeneity of inflammatory responses in FLS isolated from OA patients, revealing significant variability in MPO and IL-6 production in response to LPS and IL-6. By integrating experimental and synthetic data, the findings demonstrated the utility of clustering techniques in identifying distinct patient subgroups with variable inflammatory thresholds. These results emphasised the importance of patient-specific analysis in advancing personalised therapeutic strategies for OA.

Shapiro–Wilk tests confirmed that the data were non-parametric, supporting the use of the Kruskal–Wallis test and subsequent Dwass–Steel–Critchlow–Fligner (DSCF) comparisons. These analyses demonstrated substantial individual variability, not only between different concentrations of LPS and IL-6 but also among the patients themselves, suggesting that individual patient factors might contribute to these variations. For example, patients suffering from conditions such as high blood pressure, cholesterol, and diabetes (e.g., N4, N5, N9) demonstrated distinct MPO and IL-6 responses. These observations aligned with previous research indicating that pre-existing conditions could influence immune response dynamics, though further studies are needed to explore these associations.

The substantial variability observed among patients highlights the influence of individual factors, including underlying health conditions and medication use. Patients with comorbidities, such as diabetes and hypertension, often displayed lower MPO responses, suggesting altered inflammatory thresholds. These results aligned with previous studies linking comorbidities to immune modulation in OA [44]. By identifying patient-specific inflammatory patterns, this study laid the foundation for personalised approaches to managing OA-related inflammation, reinforcing the necessity of individualised treatments to optimise therapeutic outcomes.

Hierarchical clustering and t-distributed stochastic neighbour embedding (t-SNE) effectively identified distinct patient subgroups, revealing varied inflammatory response patterns linked to patient-specific characteristics. The robust clear performance, as indicated by ROC curves with AUC values consistently exceeding 0.7, captured dose–response variability and distinguished inflammatory thresholds. Synthetic data generation proved critical in overcoming the limitations of small sample sizes, with kNN classification and ROC curve analysis confirming alignment with experimental observations. These findings underscored the model’s utility in classifying OA patients and advancing precision medicine approaches tailored to individual inflammatory responses, complementing prior research that highlights the benefits of clustering techniques in understanding immune variability in inflammatory diseases [45].

The variability in IL-6 and MPO production, coupled with the distinct clustering of patient responses, underscores the need for personalised therapeutic approaches in OA. These findings supported the potential of using patient-specific inflammatory profiles to stratify patients and tailor treatments based on individual inflammatory thresholds. Future studies should aim to integrate these findings with molecular analyses, such as single-cell sequencing, to further elucidate the mechanisms driving variability in FLS responses [46].

Further investigations into molecular mechanisms underlying patient specific variability in FLS responses to inflammatory stimuli will be crucial in developing novel therapeutic strategies for the disease. Advanced methods such as single-cell RNA sequencing and proteomics could be employed to further elucidate the complex interactions within the synovial microenvironment and identify pathways driving variability in inflammatory responses. Additionally, investigations into the effects of medications and OA progression stages on FLS responses would offer valuable information for targeted therapeutic approaches. Expanding the dataset to include diverse patient populations is also essential to ensure the generalisability of findings across different demographic and clinical contexts. Combined, these approaches would deepen the understanding of observed variability and support the development of personalised therapeutic strategies for OA.

## 5. Conclusions

This study underscored significant variability in FLS responses to LPS and IL-6, potentially influenced by medication, disease stage, and underlying conditions. By employing both experimental and synthetic data, the analysis highlighted that inflammatory responses in OA are highly individualised, with distinct dose-dependent variability in IL-6 and MPO production. Clustering analyses identified three primary patient subgroups characterised by varying sensitivities to inflammatory stimuli, emphasising the heterogeneity of OA-associated responses. ROC curve validation confirmed the reliability of the findings, reinforcing their potential to inform patient-specific treatment strategies.

These results demonstrated the critical role of synthetic data in enhancing experimental datasets and provided a robust foundation for advancing personalised therapeutic approaches in OA. The integration of clustering techniques and dimensionality reduction underscores the feasibility of stratifying patients based on inflammatory thresholds and comorbidities, supporting targeted interventions.

Future research should aim to validate the observed patient subgroups through larger experimental datasets, enabling the identification of subgroup-specific therapeutic targets. Additionally, investigations should explore the molecular pathways underlying the variability to uncover the mechanisms driving patient-specific responses. Expanding the dataset to diverse OA populations and incorporating longitudinal analysis could refine the understanding of inflammatory progression and therapeutic outcomes. Finally, the further development of synthetic data methodologies should aim to enhance their alignment with experimental data, minimising biases and maximising translational relevance.

## Figures and Tables

**Table 1:** Kruskal–Wallis test results for biomarker (IL-6 and MPO) response to stimulants (LPS and IL-6). χ^2^ (Chi-squared), df (degrees of freedom), *p* (*p*-value), and ε^2^ (epsilon-squared).

Kruskal–Wallis				
	χ^2^	df	*p*	ε^2^
IL-6 Response to LPS	3049	3	<0.001	0.268
MPO Response to LPS	435	5	<0.001	0.0397
MPO Response to IL-6	656	4	<0.001	0.0577

**Table 2:** Kruskal–Wallis test results for biomarker (IL-6 and MPO) response to patients. χ^2^ (Chi-squared), df (degrees of freedom), *p* (*p*-value), and ε^2^ (epsilon-squared).

Kruskal–Wallis				
	χ^2^	df	*p*	ε^2^
IL-6/LPS Response to Patients	6332	6	<0.001	0.557
MPO/LPS Response to Patients	9756	7	<0.001	0.890
MPO/IL-6 Response to Patients	10,149	6	<0.001	0.893

## Data Availability

The data presented in this study are available on request from the corresponding author (due to ethical reasons).
